# Characterization of the bacterial communities on recent Icelandic volcanic deposits of different ages

**DOI:** 10.1186/s12866-018-1262-0

**Published:** 2018-09-24

**Authors:** Bo Byloos, Pieter Monsieurs, Mohamed Mysara, Natalie Leys, Nico Boon, Rob Van Houdt

**Affiliations:** 1 Microbiology Unit, Interdisciplinary Biosciences, Belgian Nuclear Research Centre, SCK•CEN, Boeretang 200, B-2400 Mol, Belgium; 2 Center for Microbial Ecology & Technology (CMET), Ghent University, Ghent, Belgium

**Keywords:** Basalt, Lava, Krafla, Iceland, Chronosequence, 16S rRNA gene amplicon sequencing, Bacterial communities

## Abstract

**Background:**

Basalt is the most common igneous rock on the Earth’s surface covering. Basalt-associated microorganisms drive the cycling and sequestration of different elements such as nitrogen, carbon and other nutrients, which facilitate subsequent pioneer and plant development, impacting long-term regulation of the Earth’s temperature and biosphere. The initial processes of colonization and subsequent rock weathering by microbial communities are still poorly understood and relatively few data are available on the diversity and richness of the communities inhabiting successive and chronological lava flows. In this study, the bacterial communities present on lava deposits from different eruptions of the 1975–84 Krafla Fires (32-, 35- and 39-year old, respectively) at the Krafla, Iceland, were determined.

**Results:**

Three sites were sampled for each deposit (32-, 35- and 39-year old), two proximal sites (at 10 m distance) and one more distant site (at 100 m from the two other sites). The determined chemical composition and metal concentrations were similar for the three basalt deposits. No significant differences were observed in the total number of cells in each flow. 16S rRNA gene amplicon sequencing showed that the most abundant classified phylum across the 3 flows was *Proteobacteria*, although predominance of *Acidobacteria*, *Actinobacteria* and *Firmicutes* was observed for some sampling sites. In addition, a considerable fraction of the operational taxonomic units remained unclassified. Alpha diversity (Shannon, inverse Simpson and Chao), HOMOVA and AMOVA only showed a significant difference for Shannon between the 32- and 39-year old flow (*p* < 0.05). Nonmetric multidimensional scaling (NMDS) analysis showed that age significantly (*p* = 0.026) influenced the leftward movement along NMDS axis 1.

**Conclusions:**

Although NMDS indicated that the (relatively small) age difference of the deposits appeared to impact the bacterial community, this analysis was not consistent with AMOVA and HOMOVA, indicating no significant difference in community structure. The combined results drive us to conclude that the (relatively small) age differences of the deposits do not appear to be the main factor shaping the microbial communities. Probably other factors such as spatial heterogeneity, associated carbon content, exogenous rain precipitations and wind also affect the diversity and dynamics.

**Electronic supplementary material:**

The online version of this article (10.1186/s12866-018-1262-0) contains supplementary material, which is available to authorized users.

## Background

Igneous rocks constitute about 95% of the Earth’s upper crust and the initial processes of colonization and subsequent rock weathering by microbial communities are still poorly understood. Microorganisms drive the cycling and sequestration of different elements such as nitrogen, carbon and other nutrients, which facilitate subsequent pioneer and plant development, impacting long-term regulation of the Earth’s temperature and biosphere. This interplay between the environment and the biosphere has big implications for the development of the Earth’s atmosphere, not only now, but also for the early Earth [1].

Young unvegetated lava deposits lack readily available nutrients and impose selective conditions on possible colonizing bacterial communities, e.g. nitrogen and carbon need to be supplied via the microorganisms’ N_2_ and CO_2_ fixating activity [1–4]. In addition, exposure to higher UV radiation, temperature fluctuations and desiccation enforce further constraints [5, 6]. Furthermore, since solidified lava cannot retain much water, early microbial colonization depends on precipitation and an exogenous source of nutrients, e.g. atmospheric trace gasses that could also serve as a source of carbon and energy [7, 8]. The best studied igneous rock formations are oceanic basalts, as about 60% of the Earth’s crust consists of basalt, and microorganisms colonizing marine basalts play an important part in the biogeochemical cycling of elements in marine water [9]. In addition, most research for initial colonization of terrestrial rocks has focused on the role of lichens [10], while work on bacteria and their role in volcanic rock weathering was only recently elucidated with *Actinobacteria*, *Proteobacteria* and *Bacteroidetes* being found to be the dominant phyla [1, 2, 5, 11–13].

Thus, relatively few data are available on the diversity and richness of the communities inhabiting successive and chronological lava flows. As there is a constant substrate input, chronosequences are formed where older, more weathered substrates are further from the substrate source, which thus serves as a proxy for time and ecosystem development. The latter can be used to study community shifts along this chronosequence to determine how this correlates with habitability development [13]. In this study, we determined and compared the microbial communities of different subsequent lava deposits around the Krafla volcanic field (Iceland) and determined element dynamics along the chronosequence of the lava flows.

## Results

### Site parameters and lava flow composition

**Fig. 1:**
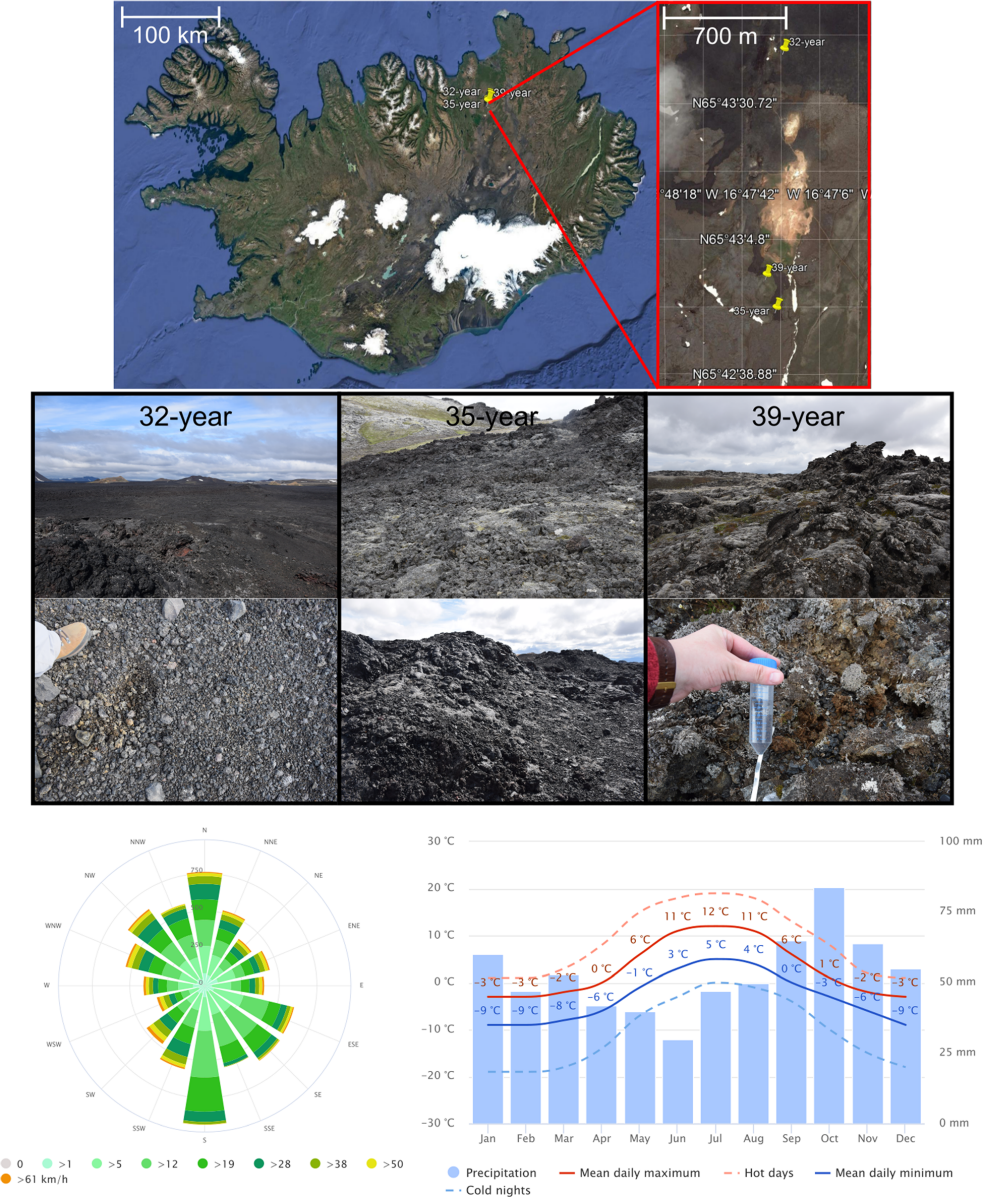
Map of the Krafla area in Iceland (Google Earth, Digitalglobe), location and pictures of the three different sampling locations on each of the fissures from the vent and lava flows produced by the eruptions of the 1975–84 Krafla Fires, and meteoblue climate (temperature, precipitation, wind speed and direction- diagrams; www.meteoblue.com)

**Fig. 2:**
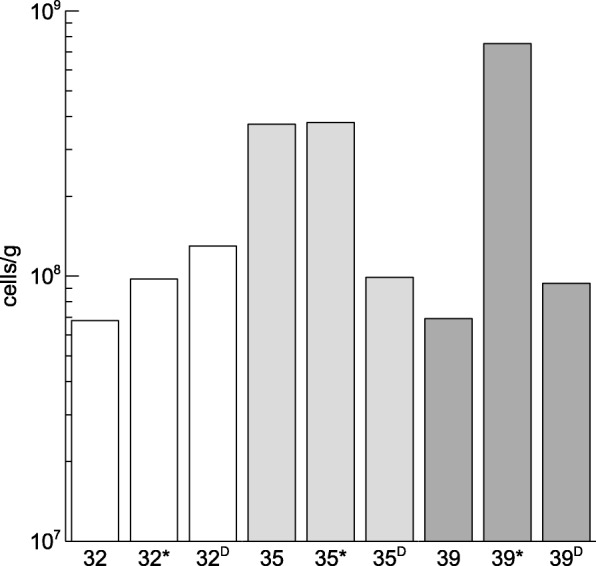
Total cell number determined by flow cytometry

The three different sampling locations represented deposits from different eruptions of the 1975–84 Krafla Fires (32-, 35- and 39-year old, respectively) and showed clear progression from poorly to fully vegetated sites. At each flow, three different points were chosen, resulting in three sites for the three different lava deposits. Two sites were in proximity to each other (10 m distance; designated as year and year*) while a third one (D) was at a distance of 100 m from the two other sites but still within the same lava deposit. Based on the meteoblue climate diagrams (www.meteoblue.com), the mean daily maximum and minimum temperature ranges between − 3° and 12 °C, and − 9 °C and 5 °C, respectively. The mean monthly precipitation ranges between 30 and 84 mm (as rain- and snowfall). The main wind direction is south. The 32-year flow is still exposed to the release of volcanic SO_2_ and H_2_S gasses and higher temperatures. No visible vegetation was observed on the 32-year flow. The 35-year flow showed lichen formation while the 39-year old flow already showed the formation of grasses and mosses (Fig. 1). The total number of cells determined by flow cytometry showed that the three sampling sites within the three flows contained between 6.80 × 10^7^ and 7.54 × 10^8^ cells/g (Fig. 2).

### Basalt composition

The determined chemical composition and metal concentrations were similar for the three flows (Tables 1 and 2).

**Table 1:** Chemical composition (wt%) of the different basalts^a^

	32-year	35-year	39-year
SiO_2_	49.41	49.15	49.42
Al_2_O_3_	13.18	13.24	13.38
Fe_2_O_3_	15.49	15.06	14.19
MnO	0.23	0.23	0.22
MgO	5.64	5.76	5.88
CaO	9.96	9.50	9.97
Na_2_O	2.33	2.30	2.34
K_2_O	0.36	0.32	0.33
TiO_2_	2.00	1.99	1.84
P_2_O_5_	0.23	0.22	0.22
FeO	12.30	9.00	11.10
LOI^b^	0.07	2.19	1.47
Total	98.90	99.96	99.25

^a^Uncertainty of the measurement is 0.01%; ^b^LOI: loss on ignition

**Table 2:** Metal concentration (ppm) in the different basalts^a^

	32	35	39
Zn	130	130	120
Cu	120	160	130
Ni	80	80	90
Cr	120	120	140
V	438	420	405
Ba	89	90	89
Sc	44	44	43
La	10.3	10.1	10.6
Ce	25.1	24.6	25.1
Nd	16	15.9	15.5
U	0.2	0.2	0.2
Th	0.8	0.8	0.8
Pb	bd	bd	bd
Nd	16	15.9	15.5
Zr	118	120	119
Y	30	30	29
Sr	151	147	148
Rb	9	7	7

^a^Uncertainty of the measurement is 0.1%; bd: below detection limit

### Taxonomic distribution within each flow and comparison between the different flows and sites

**Fig. 3:**
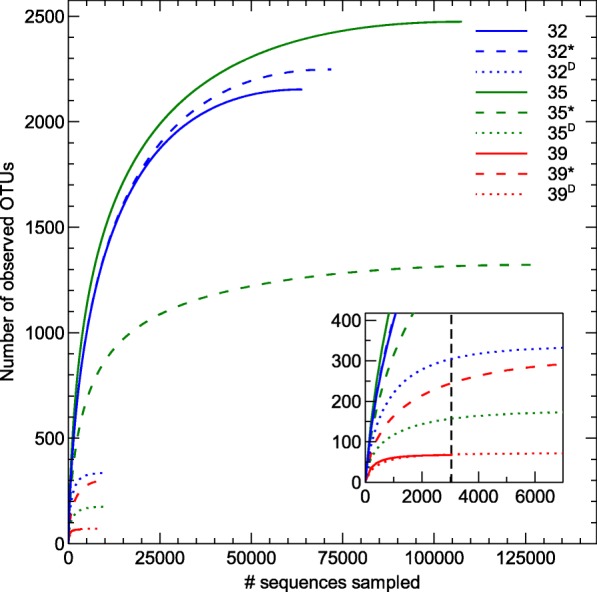
Rarefaction curve of OTU diversity for each sample

Subsampling was performed based on the lowest amount of reads obtained over the nine different samples, i.e., 32-year sample with a coverage of 3060 reads (Fig. 3). Such a subsampling procedure allowed us to make a fair comparison between the diversity indices for all samples, e.g. avoiding a potential higher diversity due to a higher sequencing coverage.

**Fig. 4:**
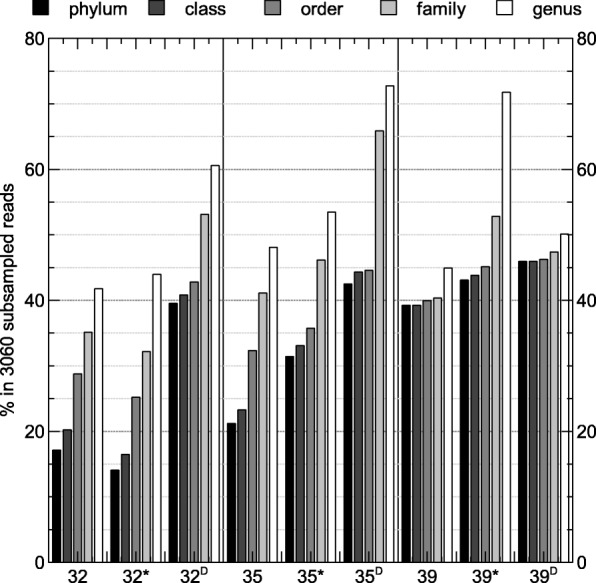
Number of unclassified reads for different taxonomic ranks

**Fig. 5:**
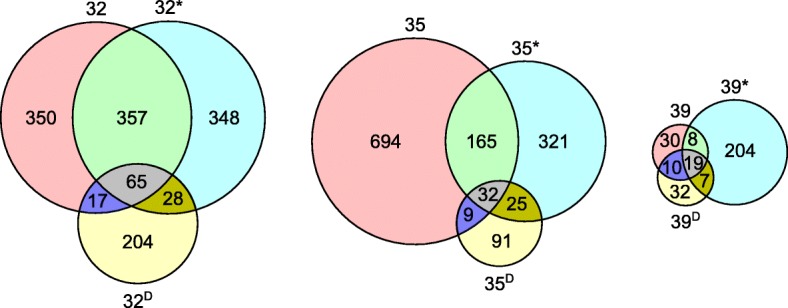
Weighted Venn diagram representing the number of OTUs present in the 32-, 35- and 39-year old flow as well as the number of unique and shared OTUs for the sampling sites within one flow

Taxonomic profiling indicated that a large fraction of the operational taxonomic units (OTUs) could not be classified (Fig. 4; Additional file 1: Table S3). At the level of taxonomic families, sites 32, 32* and 32^D^ contained about 35%, 52% and 53% unclassified sequences, respectively. For the 35-year old flow, 35 and 35* contained 41 and 46% unclassified sequences while 35^D^ contained 66% unclassified sequences. The three sites within the 39-year old flow (39, 39* and 39^D^) contained 40%, 53% and 47% unclassified sequences, respectively. An additional overview of the classified and unclassified number of raw reads, OTUs, single-read OTUs as well as OTUs contributing more than 1% to the taxonomic distribution is given in Table 3. Nevertheless, none of the OTUs identified in the samples were observed in the negative control, which was comprised of sterile water that was subjected to the same processing pipeline as the basalt samples. The major OTUs (relative abundance above 1%) constituted 1 to 18% of all OTUs, the single-read OTUs constituted 0 to 56% of all OTUs. The OTUs shared by the different sampling sites within each flow clearly differed (Fig. 5).

**Table 3:** Overview of the number of classified and unclassified raw reads, OTUs, single-read OTUs and OTUs with a relative abundance above 1%

	32	32*	32^D^	35	35*	35^D^	39	39*	39^D^
Classified									
# reads	1792	1725	1221	1600	1436	851	1695	881	1539
%	59.03	56.82	40.22	52.70	47.30	28.03	55.83	29.02	50.69
# OTUs	332	323	156	336	249	87	52	103	48
# single-read OTUs	170	148	29	149	103	23	0	30	3
# OTUs > 1%	8	11	3	6	7	5	10	5	9
Unclassified									
# reads	1268	1335	1839	1460	1624	2209	1365	2179	1521
%	41.77	43.97	60.57	48.09	53.49	72.76	44.96	71.77	50.10
# OTUs	457	475	158	564	294	70	15	135	20
# single-read OTUs	263	295	28	298	124	9	0	43	1
# OTUs > 1%	2	4	7	1	8	18	2	9	3

**Fig. 6:**
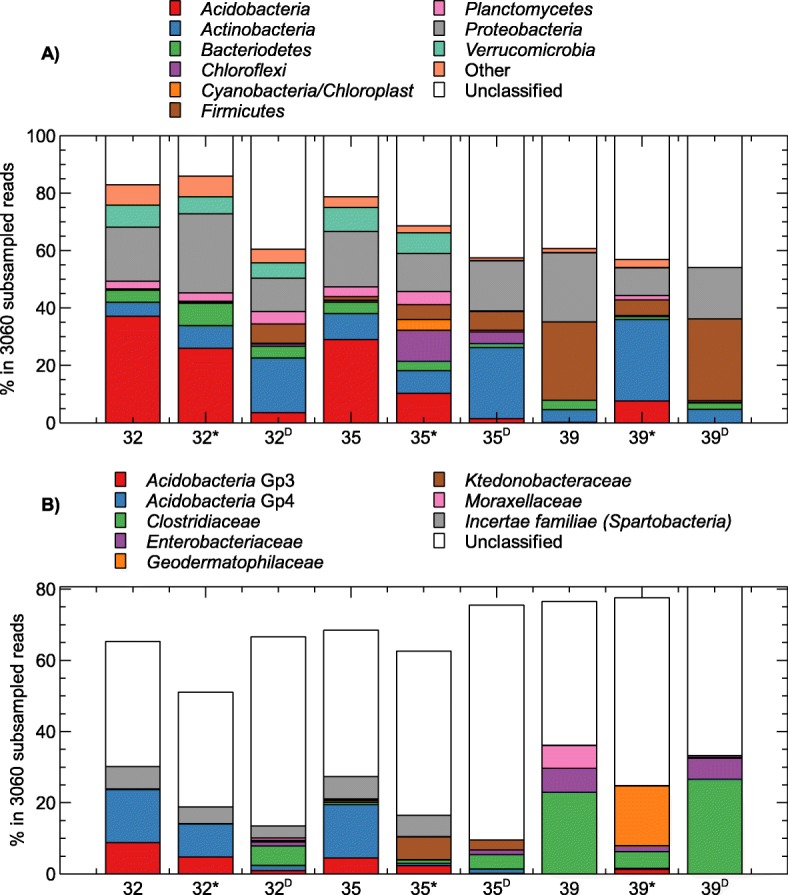
Relative abundance of the bacterial phyla (**a**) and major bacterial families (**b**) in the 32-, 35- and 39-year old flows based on 16S rRNA gene sequencing data. Only families with a relative abundance above 2% in at least one sample are shown

**Fig. 7:**
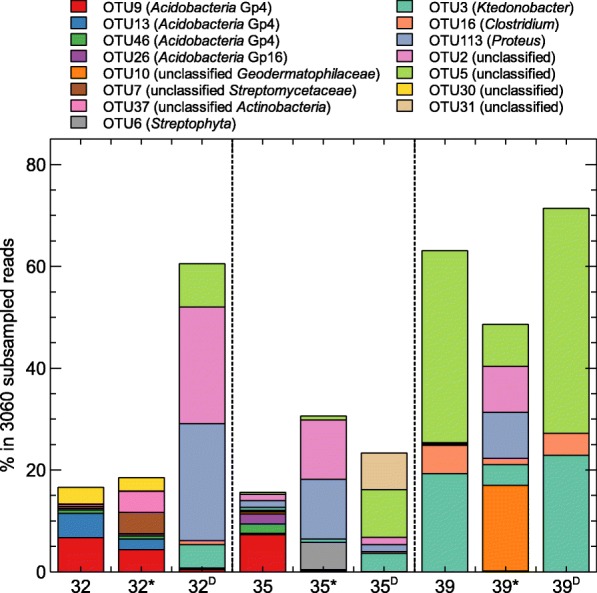
The three most abundant OTUs in each sampling site and their respective abundance in the other samples

In the 32-year old flow, the most abundant classified phylum across the three sites was *Proteobacteria*, although site 32 and 32^D^ were predominated by *Acidobacteria* (mainly Gp4) and *Actinobacteria*, respectively (Fig. 6). The most abundant OTU belonged to Gp4 (*Acidobacteria*; OTU9) for site 32 (6.7%) and 32* (4.3%), and was unclassified OTU2 (24.6%) for the more distant site (Fig. 7).

In the 35-year old flow, the most abundant classified phylum across the three sites also was *Proteobacteria*, although site 35 was predominated by *Acidobacteria* (Gp4 family) and site 35^D^ by *Actinobacteria* (Fig. 6). The most abundant OTU belonged to Gp4 (*Acidobacteria*; OTU9) for site 35 (7.3%), was unclassified OTU2 for site 35* (11.7%) and unclassified OTU5 for site 35^D^ (9.4%) (Fig. 7).

In the 39-year old flow, the most abundant classified phylum across the three sites was *Firmicutes* (mainly *Clostridiaceae*), although site 39* was predominated by *Actinobacteria* (mainly *Geodermatophilaceae*) (Fig. 6). The most abundant OTU in site 39 and 39^D^ was OTU5 (37.7% and 44.2%, respectively), and belonged to *Geodermatophilaceae* for site 39* (OTU10; 16.8%) (Fig. 7).

One hundred and five OTUs were shared between at least one sampling site of the 32-, 35- and 39-year old flow (Additional file 1: Table S3). Three were common to all samples and classified as *Clostridium* (OTU16), *Propionibacterium* (OTU22) and *Bradyrhizobium* (OTU48). Next to the OTUs shared by all three flows, additional OTUs were shared when flows were pairwise compared. The 32- and 35-year old flows additionally shared seven OTUs classified as *Acidobacteria* (OTU9), *Actinobacteria* (OTU65, OTU146 and OTU170), *Proteobacteria* (OTU67 and OTU79) and unclassified (OTU68). The 32- and 39-year old flows additionally shared one unclassified OTU (OTU302). The 35- and 39-year old flows additionally shared six OTUs classified as *Bacteroidetes* (OTU195), *Firmicutes* (OTU176), *Proteobacteria* (OTU113, OTU262 and OTU405) and unclassified (OTU5). The three most abundant OTUs in each sample and their abundance in the other samples indicated the high abundance of a few unclassified OTUs, especially in the 39-year old flow (Fig. 7).

### Diversity and NMDS analysis between sites and comparison

Rarefaction curves (Fig. 3) were visualized to indicate if the level of subsampling adequately represented the bacterial diversity in the samples. This showed that subsampled data only fully characterized sampling sites 32^D^, 35^D^, 39 and 39^D^. Therefore, results need to be interpreted cautiously. Nevertheless, alpha diversity (Shannon, inverse Simpson and Chao) calculated on the subsampled and complete data set gave the same conclusion (Table 4, Additional file 2: Table S2). Inverse Simpson and Chao index did not significantly differ between the 32-, 35- and 39-year old flow. Shannon differed significantly between the 32- and 39-year old flow (*p* < 0.05). Homogeneity of molecular variance (HOMOVA) and analysis of molecular variance (AMOVA) indicated no significant differences between the bacterial community structures of the 32-, 35- and 39-year old flow.

**Table 4:** Diversity indices of bacterial communities in the sampled sites

Location	Inverse Simpson	Shannon	Chao
32	3.9	2.2	69
32*	29.0	3.9	183
32^D^	15.3	4.3	344
35	82.0	5.6	1443
35*	113.7	5.7	1600
35^D^	116.3	6.0	1454
39	45.1	5.1	776
39*	5.3	2.5	67
39^D^	17.9	3.9	305

**Fig. 8:**
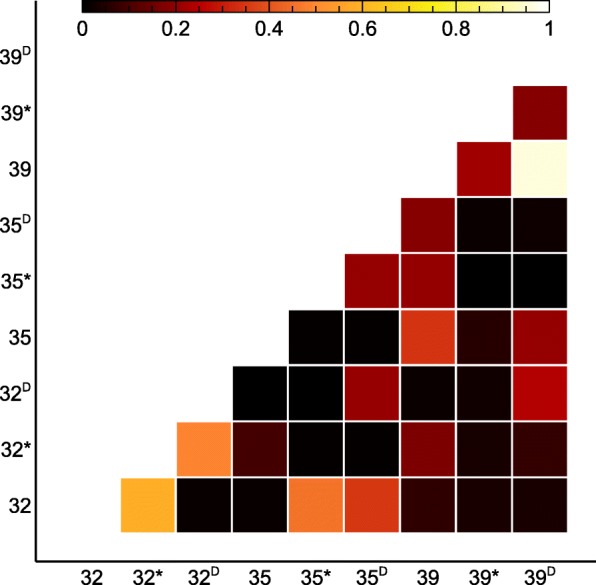
Heat map of the Yue & Clayton measure of dissimilarity

**Fig. 9:**
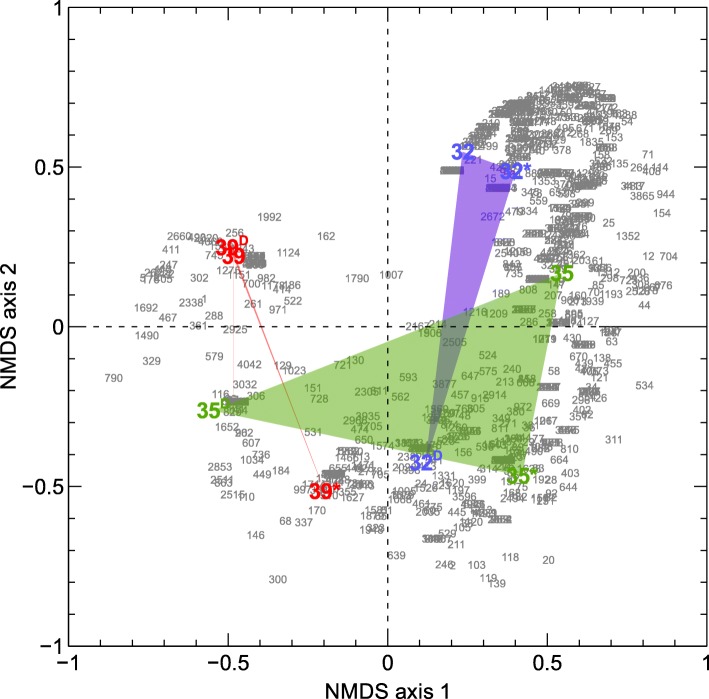
Nonmetric multidimensional scaling (NMDS) analysis of the OTUs of the bacterial communities in the 32-, 35- and 39-year old flows

Beta-diversity analysis (Yue & Clayton) indicated that the proximal 32-year old sampling sites as well as the 39 and 39^D^ sampling sites were highly similar (Fig. 8). Likewise, nonmetric multidimensional scaling (NMDS) analysis indicated that the proximal 32-year old sampling sites were similar as well as the 39 and 39^D^ sites, since these ordinated closer together (Fig. 9). The 35-year old sites do not cluster together, possibly indicating that differences for the 35-year flow are more pronounced than for the other flows. Twenty-eight OTUs significantly impacted the downward movement along NMDS axis 2 (with 12 being unclassified). Seventeen OTUs significantly impacted the leftward movement along NMDS axis 2 (with 8 being classified as *Proteobacteria*). In addition, age significantly (*p* = 0.026) influenced the leftward movement along NMDS axis 1.

## Discussion

Lava deposits from different eruptions of the 1975–84 Krafla Fires (32-, 35- and 39-year old, respectively) were sampled to determine their bacterial communities. The three different sampling locations, which showed clear progression from poorly to fully vegetated, contained between 6.80 × 10^7^ and 7.54 × 10^8^ cells/g (Fig. 2). Similar cell numbers were found in the rock-soil boundary zones in vegetated sites (both lichens and mosses, and trees) surrounding the Skorradalur lake in Iceland [14]. In contrast, cell number concentrations in recent volcanic deposits studied by Kelly et al. [11] were one log lower.

Taxonomic profiling indicated that a large fraction of the operational taxonomic units (OTUs) could not be classified (Fig. 4; Additional file 1: Table S3). Previously, Lukito et al. [15] also observed that 70% of the OTUs found in Icelandic volcanic glass were unclassifiable. This large fraction of unclassified OTUs could be due to the fact that these environments are not well characterized or because of processing artifacts during DNA amplification as only a low amount of DNA was extracted from the samples (Additional file 3: Table S1). Nevertheless, none of the OTUs identified in the samples were observed in the negative control, which was comprised of sterile water that was subjected to the same processing pipeline as the basalt samples. The low yield could result from natural DNA-binding components, such as humic acids from andosols formation within the older depositions, and DNA sequestration in a hard mineral matrix [15–17].

The most abundant phyla (*Acidobacteria*, *Actinobacteria, Firmicutes* and *Proteobacteria*) identified in our samples have already been associated with core basalt communities [3, 18, 19]. Furthermore, *Actinobacteria* members have been shown to enhance basalt weathering, resist desiccation and possess oligotrophic growth capabilities [5, 14, 20, 21]. They are seen as the key community within the basalt environment as they could possibly act as a C and N source and leach P and S [18, 22]. The different flows also contained *Verrucomicrobia* (Additional file 1: Table S3), which have been isolated from the Arctic environment and were found to be associated with basalt environments where they greatly contributed to basalt weathering [23]. All flows contained *Planctomycetales*, *Rhizobiales*, *Rhodospirillales* and *Sphingomonadales* (Additional file 1: Table S3)*,* which have been associated with basalt communities in different volcanic environments [24–27], deep seafloor sediments and lava formed soil [9, 28]. The heterotrophic capacity of the microbial community on basalt has been proposed to take part in obsidian weathering [29] and could take the available carbon either from the rock (via snowmelt or rain) or through other members of the community [3, 19]. In addition, other community members (found in all three flows) such as those belonging to *Chloroflexi*, and more specific *Ktedonobacter* (Additional file 1: Table S3), have also already been associated with the seafloor basalt of Arctic ridges [19] and a volcanic ice cave ecosystem at Mt. Erebus, Antarctica [30]. Other members (found in all three flows) such as *Chitinophagaceae* (*Bacteroidetes*) were not yet found to be associated with basalt communities. They probably will stay latent but can flourish when cellulytic substrates substrates become available [31].

Although the Chao index did not significantly differ between the 32-, 35- and 39-year old flow, they were significantly higher than those measured in seafloor basalts (ranging from 12 to 157) [16] as well as in recent Hawaiian volcanic deposits [5]. Also Kelly et al. [11] reported lower numbers for the basaltic Fimmvörðuháls lava flow (Eyjafjallajökull, Iceland) with Chao ranging from 20 to 120 and Shannon index ranging from 1.4 to 2.49. This could indicate that the flows sampled in this study differ from other basaltic environments and are richer in diversity.

Almost no studies have investigated the microbial communities and dynamics present in non-vegetated sections of chronosequences, except for Teixera et al. [32] who showed that in glacier forelands an increase in richness is correlated with increased microbial biomass and activity. Our results indicated that the (relatively small) age difference of the deposits appeared to impact the bacterial community (NMDS); however, this analysis was not consistent with AMOVA and HOMOVA, indicating no significant difference in community structure. The combined results drive us to conclude that the (relatively small) age differences of the deposits do not appear to be the main factor shaping the microbial communities. Probably other factors such as spatial heterogeneity, associated carbon content, exogenous rain precipitations and wind also affect the diversity and dynamics. The impact of spatial heterogeneity can already be seen by comparing the two proximal with the more distant site of the 32-year old flow. In addition, factors such as changes in the mineral substrate, porosity, pH and surface characteristics have also been shown to influence bacterial community composition of terrestrial volcanic rocks [33–35]. For instance, rock mineralogy will affect weathering rates, nutrient availability and albedo. The latter influences the temperatures that microbes may experience within the rock [6, 36, 37].

## Conclusion

Identification of the bacterial communities present on lava deposits from different eruptions of the 1975–84 Krafla Fires (32-, 35- and 39-year old, respectively), with different vegetation, showed that the (relatively small) age differences of the deposits do not appear to be the main factor shaping the microbial communities.

## Methods

### Sample sites and sampling

Samples were taken from three different locations on the Leirhnjúkur fissures formed around Krafla, Iceland from the 1975–84 Krafla Fires. During this period, there was significant periodic rifting and faulting along the plate boundary, which was confined to a single system around Krafla. Both rifting episodes included a series of nine small (< 0.2 km^3^) effusive eruptions that occurred on fissures within the Krafla caldera volcano as well as on the nearby sectors of the fissure swarms [13]. On 27 July 2016, 9 sample sites were defined, three on each of the three lava flows (32-, 35- and 39-year old) originating from the fissure swarms (Fig. 1). The three flows were clearly different in vegetation ranging from almost none (youngest) to fully (oldest) vegetated and were relatively accessible for sampling. Within each flow, three sites were selected. Two sites were in proximity to each other (10 m distance; designated as year and year*) while a third one (^D^) was at a distance of 100 m from the two other sites but still within the same lava deposit (Fig. 1). Coordinates of the 32-, 35- and 39-year old lava flows are 65°43’39.828’ ’N-16°47’23.3372’ ’W, 65°42’50.5332’ ’N-16°47’26.7576’ ’W and 65°42’56.8872’ ’N-16°47’31.3908’ ’W, respectively.

About 20 g of smaller sized rocks and soil material was sampled at 5 cm below the upper part of the deposits with a sterilized spoon and collected in a 50 ml sterile falcon tube (CELLSTAR® Centrifuge Tubes, Polypropylene, Sterile, Greiner Bio-One, Belgium). These tubes were sealed in three layers of plastic bags and transported at ambient temperature to the laboratory for analysis (see below). The time between collection and analysis was 7 days.

### Composition analysis

Five grams of basalt rock material, with a mesh size below 200, were prepared and used to analyze the composition of the different basalt rocks, with lithium metaborate/tetraborate fusion and inductively coupled plasma optical emission spectrometry (ICP-OES)/inductively coupled plasma mass spectrometry (ICP-MS) analysis, and titration for FeO (Actlabs, Canada) (Tables 1 and 2).

### Flow cytometry

Five grams of sample was added to 50 ml Butterfield’s Phosphate Buffer (0.25 M KH_2_PO_4_ in distilled water, adjusted to pH 7 with 1 M NaOH) and incubated at 30 °C in static conditions. After overnight incubation, part of the buffer solution was analysed with flow cytometry to analyse the total cell numbers (SYBR Green; SG) [38]. Samples were stained according to optimized procedures [39, 40]. Samples were prepared by diluting the sample (to obtain an event rate between 200 and 2000 events/μl) in 0.2 mm filtered mineral water. Next, SG dye (Sigma Aldrich; final concentration of 1×) was added and cell suspensions were incubated at 35 °C for 13 min. Stained bacterial suspensions were analysed on an Accuri C6 (BD, Erembodegem) with a blue (488 nm, 20 mW) and red (640 nm, 14.7 mW) laser, which was calibrated according to the manufacturer’s recommendation. Standard optical filters were used and SG was detected in FL-1 (530/30 nm) with the blue laser. A quality control with 6- and 8-peak fluorescent beads (by manufacturer BD, Erembodegem) and a cleaning cycle were performed prior to experiments to assess both the accuracy (bead count and position) and the cleanliness. Samples were analysed using the Accuri C6 software (version 1.0.264.21).

### DNA extraction

To limit DNA extraction bias, two different protocols were used. DNA was extracted from 1 g basalt using the bead-beating cetyl trimethylammonium bromide (CTAB) phenol extraction protocol [41] and the FastPrep protocol [42] without the last Sepharose 4B based purification step [43]. Afterwards, extracted DNA was dissolved in 1xTE buffer and stored at -20 °C.

### Illumina sequencing

The DNA concentration was quantified by the Quantifluor dsDNA sample kit on a multi-detection system (Promega, Leiden, the Netherlands). High-throughput amplicon sequencing of the V3-V4 hypervariable region [44] was performed with the Illumina MiSeq platform according to the manufacturer’s guidelines at LGC Genomics GmbH (Berlin, Germany). Sequences were preprocessed using the OCToPUS pipeline combining various preprocessing algorithms [45]. First, both forward and reverse reads are quality checked via looking at k-mer frequency to identify potential false k-mers using the Hammer algorithm implemented in the SPAdes tool. Next, the contigs were created by merging the paired-end reads using the heuristic based on the difference in Phred quality scores of both reads using an updated version of the mothur command “make.contigs” as described in [46]. These contigs were aligned to the SILVA database [47], followed by removing those contigs having an ambiguous base, homopolymer longer than 8 nt, or a length below 400, and those incompliant with the targeted region within the 16S rRNA gene using the mothur commands “align.seqs” and “screen.seqs”, respectively. Next, the aligned sequences were filtered and dereplicated using the mothur commands “filter.seqs” and “unique.seqs”. Secondly, sequencing errors were removed using the IPED algorithm - dedicated to denoise MiSeq amplicon sequencing data [45]. Chimera were detected using the de novo mode of the CATCh algorithm [46]. Creation of the operational taxonomic units (OTUs) was performed using UPARSE ([48] with default parameters (v7.0.1001_i86linux32 – commands sortbysize, cluster_otus, and usearch_global) [42, 45, 46, 49–52]. The datasets generated and analyzed during the current study are available in the NCBI Sequence Read Archive (SRA) repository (SRP106138).

### Statistics

The datasets generated by the two DNA extraction methods were combined for further analyses. Subsampling was performed using the the mothur command “sub.sample” using the amount of reads of the smallest sample. Alpha diversity indices (Chao, Shannon, and inverse Simpson) were calculated using the mothur command “*summary.single”*. The beta-diversity between the samples was assessed using the Yue & Clayton measure of dissimilarity (mothur command “*dist.shared*”) and non-metric multidimensional scaling (NMDS) plots (mothur command “*nmds*”). For the NMDS plot, statistical significant correlation with the coordinates was indicated (*p* < 0.05) (mothur *corr.axes* command). The structure of the microbial populations was assessed using the analysis of molecular variance (mothur command “*amova*”) and homogeneity of molecular variance (mothur command *“homova*”). Statistical comparison of indices was performed using a one-way ANOVA analysis followed by a post-hoc Tukey test (including multiple testing).

## Additional files

Additional file 1: **Table S3.** Overview of the subsampled dataset generated in this study. (XLSX 109 kb)

Additional file 2: **Table S2.** Diversity indexes for each of the sampled sites calculated for the complete and subsampled data. (DOCX 20 kb)

Additional file 3: **Table S1.** DNA yield obtained from the bead-beating cetyl trimethylammonium bromide (CTAB) phenol extraction [41] and the FastPrep [42] method from 1 g of sample. (DOCX 19 kb)

## Abbreviations

AMOVA: Analysis of molecular variance
CTAB: Cetyl trimethylammonium bromide
HOMOVA: Homogeneity of molecular variance
ICP-MS: Inductively coupled plasma mass spectrometry
ICP-OES: Inductively coupled plasma optical emission spectrometry
NMDS: Nonmetric multidimensional scaling
OTU: Operational taxonomic unit
