# Supplementary material for: Characterization of the bacterial communities on recent Icelandic volcanic deposits of different ages
Source: BMC Microbiol. 2018 Sep 24;18:122. doi: 10.1186/s12866-018-1262-0 (PMC6154810; doi:10.1186/s12866-018-1262-0)
Supplement: Supplementary file 2 — Table S2. Diversity indexes for each of the sampled sites calculated for the complete and subsampled data. (DOCX 20 kb) [file 12866_2018_1262_MOESM2_ESM.docx]

| Table S2: Diversity indexes for the sampled sites based on the complete data set (non-rarefied) | | | | | | |
| --- | --- | --- | --- | --- | --- | --- |
| Location | Inverse Simpson | | | Shannon | | Chao |
| 32 | 89.2 | | | 5.9 | | 2153 |
| 32* | 109.6 | | | 6 | | 2248 |
| 32^D^ | 14.8 | | | 4.3 | | 335 |
| 35 | 111.8 | | | 6.3 | | 2474 |
| 35* | 47.6 | | | 5.3 | | 1322 |
| 35^D^ | 28.6 | | | 3.9 | | 175 |
| 39 | 5.3 | | | 2.5 | | 67 |
| 39* | 17.8 | | | 4 | | 299 |
| 39^D^ | 3.9 | | | 2.2 | | 71 |
|  | |  |  | |  |  |
