# Supplementary material for: Characterization of the bacterial communities on recent Icelandic volcanic deposits of different ages
Source: BMC Microbiol. 2018 Sep 24;18:122. doi: 10.1186/s12866-018-1262-0 (PMC6154810; doi:10.1186/s12866-018-1262-0)
Supplement: Supplementary file 3 — Table S1. DNA yield obtained from the bead-beating cetyl trimethylammonium bromide (CTAB) phenol extraction [41] and the FastPrep [42] method from 1 g of sample. (DOCX 19 kb) [file 12866_2018_1262_MOESM3_ESM.docx]

| Table S1: DNA yield (ng) obtained from 1 g of basalt sample. | | | | | |
| --- | --- | --- | --- | --- | --- |
| Location | | bead-beating CTAB phenol | | FastPrep | |
| 32 | | 912.00 | | 182.85 | |
| 32* | | 290.40 | | 259.05 | |
| 32^D^ | | 151.20 | | 4.94 | |
| 35 | | 185.70 | | 98.28 | |
| 35* | | 474.00 | | 56.66 | |
| 35^D^ | | 0.20 | | 1.98 | |
| 39 | | 6.00 | | 6.45 | |
| 39* | | 16.50 | | 11.91 | |
| 39^D^ | | 0.74 | | 1.37 | |
|  |  | |  | |  |
